# Teaching point-of-care ultrasound using a serious game: a randomized controlled trial

**DOI:** 10.1186/s12909-023-04964-0

**Published:** 2023-12-19

**Authors:** Tycho Olgers, Jelle Rozendaal, Sanne van Weringh, Rachel van de Vliert, Ranek Laros, Hjalmar Bouma, Jan ter Maaten

**Affiliations:** 1 Department Internal Medicine, University of Groningen, University Medical Center Groningen, Groningen, 9700 RB The Netherlands; 2 Faculty of medical sciences, University of Groningen, Postbus 72, Groningen, 9700 AB The Netherlands

**Keywords:** POCUS, Serious game, Ultrasound education, Internal medicine

## Abstract

**Background:**

Point-of-care ultrasound (POCUS) is an important diagnostic tool for internists. However, there are important barriers in learning POCUS, including lack of practice time and lack of experts for supervision. Alternative learning tools may assist in overcoming these barriers. A serious game is being developed specifically for teaching ultrasound. In this study, we assessed the use of a serious game in learning POCUS.

**Methods:**

Ultrasound-native medical students were randomly assigned to the intervention group (N = 27) or the control group (N = 26). Both groups performed a real ultrasound on a volunteer after a brief introduction, but the intervention group played a serious game in advance. The endpoints were the assessments of the videos by experts (scoring quality of the probe movements) and the research team (counting probe movements), and probe movements measured with an accelerometer.

**Results:**

The intervention group completed the exam faster (247 s vs. 347 s, *p* = 0.006 (95% CI: [30.20;168.80]) and lifted the probe less frequently from the model (0.54 vs. 3.79, *p* = 0.001 (95% CI: [1.39;5.11]). Also, we found an in-game learning effect between levels, showing a 48% decrease in total playing time (*p* < 0.001), 36% reduction in attempts per coin (*p* = 0.007), a 33% reduction in total probe distance (*p* = 0.002), and a 61% decrease in contact moments (*p* < 0.001). However, there was no significant difference in expert score between the two groups.

**Conclusion:**

The serious game ‘Underwater’ is a fun and useful addition to traditional bedside ultrasound learning, which also may overcome one of the most important barriers in learning ultrasound: lack of supervised practice time. We show that the game improves real-practice probe handling with faster and more goal-oriented probe movements.

**Supplementary Information:**

The online version contains supplementary material available at 10.1186/s12909-023-04964-0.

## Introduction

Point-of-care ultrasound (POCUS) is an important diagnostic tool for medical specialties including internal medicine [1, 2]. Several training programs exist that describe the core applications for these specialties, the specific competences needed and the minimum requirements to become qualified for reliable clinical decision-making using POCUS [3–5]. Learning POCUS requires adequate probe handling which means making logical 3D movements with the probe resulting in changes on a 2D ultrasound screen. This visuospatial orientation is difficult for beginners. Several barriers exist in learning POCUS in real practice and the most prevalent ones are lack of training, lack of experts for supervision, lack of time, and lack of an ultrasound machine [6, 7]. Serious gaming has the potential to bring new opportunities to overcome these barriers. Other specialties already successfully make use of serious games to teach technical skills [8, 9]. These games have several beneficial effects including optimizing precious supervised hands-on time, training opportunities independent of the presence of patients or availability of an expensive simulation room or manikin, and the possibility of making mistakes without adverse effects on patient care. They also increase learner satisfaction and improve knowledge retention, especially when a competition environment is created [10]. Many features of a high-quality learning environment are also found in video games like having a clear learning goal, an opportunity for practice and reinforcement, monitoring of progress and adaptation to level of the learner [11].

A serious game for teaching ultrasound is under development but has not yet been used in ultrasound training programs [12]. In short, the setup of the serious game ’Underwater’ consists of a 3D printed ultrasound probe with a stylus pen inside and a touchpad connected to a laptop with the installed game. The purpose is to collect coins in an underwater world by maneuvering the 3D-printed probe to learn ultrasound probe-handling and visuospatial orientation. We previously described the development and first steps in validating the serious game “Underwater” [13]. The aim of this study was to investigate the effect of a serious game in learning POCUS on real subjects.

## Methods

### Study design

We have performed a randomized controlled trial to assess the effects of a serious game training session on the performance of a POCUS examination on healthy volunteers. We recruited a group of third year medical students at the University of Groningen. Data were collected during a time span of two weeks in April 2021 at the University Medical Center Groningen. Participants were eligible if they had no previous ultrasound experience. They were recruited via a WhatsApp group for third year medical students (N = 256) with an invitation to apply for the research by an online Google form. All potential participants who responded to the Google Forms were invited to enroll themselves in an online document with time slots. In this document there were several possibilities for days and time slots. Every day the intervention and control group were in succeeding order: first intervention, then control, then intervention again. In this online document it was not named which time slot belonged to the intervention or control group, thereby blinding the participants for the group in which they enrolled themselves. All participants provided written informed consent. The study exam was recorded with a smartphone mounted to a standard, showing both the probe movements (only hand and probe) and the corresponding ultrasound image, in order to make the videos available for review afterwards by two independent experts and the research team. A coding list was made to assure anonymity during review of the videos. A target population of at least 40 participants was chosen based on similar experiments [14]. The study has been performed in accordance with the Declaration of Helsinki and the protocol was approved by the Medical Ethics Review Board of the University Medical Center Groningen (METc UMCG: 2019/656).

### Study protocol

**Fig. 1:**
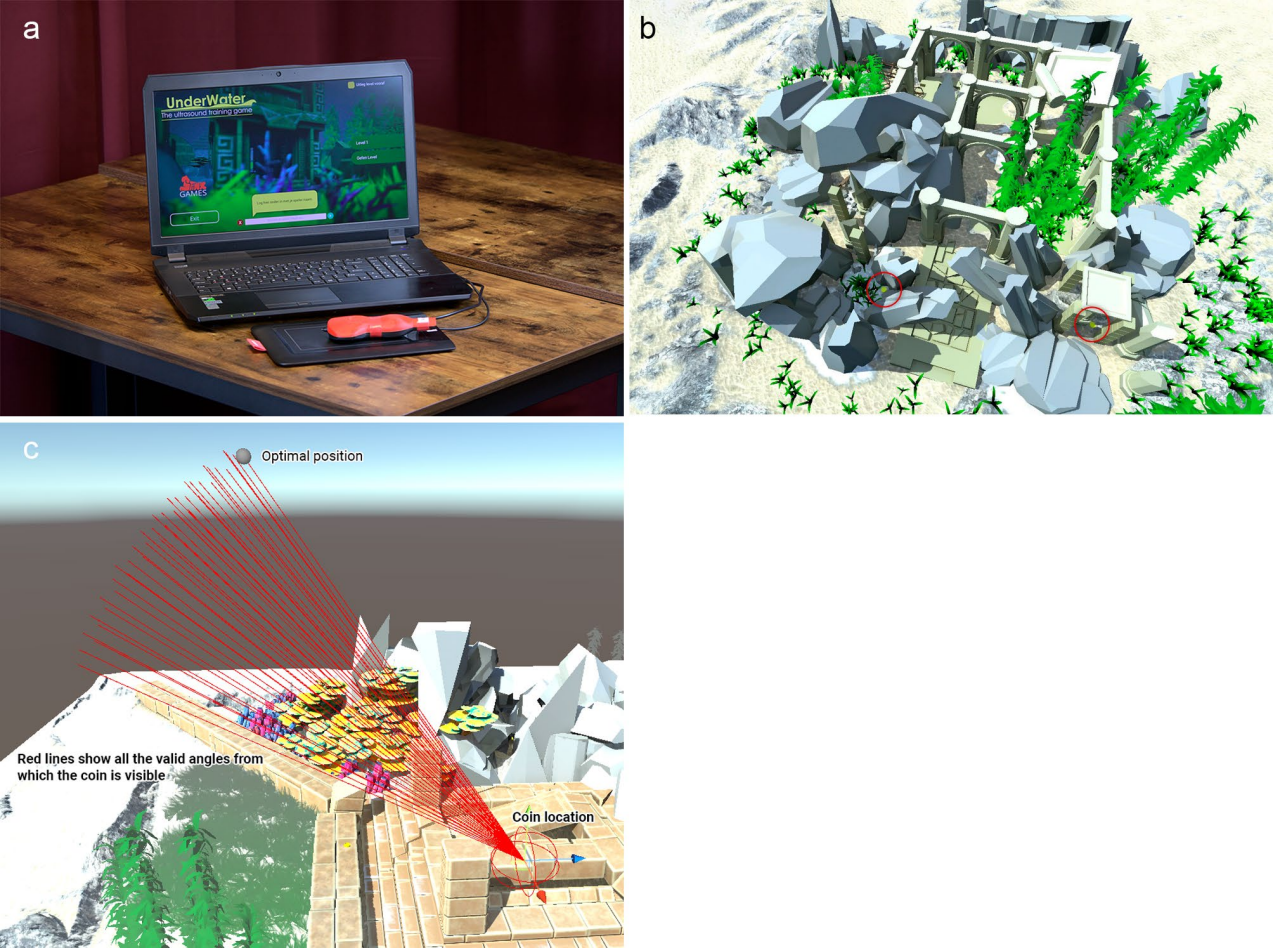
setup of the game. (**1A**) Game setup (**1B**) visible coins. (**1C**) optimal probe position and other valid angels to visualize coins

All participants received a standardized 8-minute video instruction developed by the researchers, including a video made by the ultrasound device developer (Sonosite) explaining the inferior vena cava (IVC) IVC exam, with an overview of the anatomy of the aorta and the IVC (this anatomy has already been taught previously in medical school) and an instruction for visualizing these structures [15]. The intervention group played the serious game ‘Underwater’ (Fig. 1) for 30 min after the instruction video. Next, all participants received a short standardized instruction about the exam in which they were asked to perform ultrasound imaging of the aorta, followed by the IVC and the inlet of the IVC into the right atrium (RA). They were encouraged to tell the instructors what structures were visualized on screen and when they were satisfied with the image. An anatomical image of the IVC and aorta was available for them. Participants were not able to observe each other while gaming or performing ultrasounds, due to a carefully made schedule to minimize information bias. Moreover, the assisting researcher and model did not know if the participant was in the intervention or in the control group. Ultrasound was performed with a Sonosite IVIZ portable device, on two models with a similar posture.

### Serious game ’underwater’

The setup of the serious game ’Underwater’ consists of a 3D printed ultrasound probe with a stylus pen inside and a touchpad connected to a laptop with the installed game. The setup was provided by the game manufacturer, but this company was not in any way involved in the design of the study or the conduction of the experiment. The game represents an underwater world where one is supposed to look for coins and to collect them’. The coins are collected by pointing the cursor exactly on the coin for at least one second. There is just one coin visible at any moment and coins have different levels of difficulty depending on where they are hidden, for example (partly) under objects. A level consists of 10 coins. The cursor on screen is moved by operating a simulated 3D printed probe across a touchpad using the same movements as a real ultrasound probe: rocking, tilting, sliding, and rotation. The intervention group started playing at level 1, and after completion they continued playing at other levels for approximately 20 min. They followed the protocol by playing level 1 for a second time. By repeating level 1 at a standardized level (meaning that the coins were hidden at the same spot), we were able to measure changes in time and in the quality of the movements, and therefore we could distinguish a learning curve within the game itself. An image of the setup, an in-game overview and visualization of the valid routes are provided in Fig. 1A-C.

### Data collection

Basic characteristics of the participants were collected using questionnaires to reveal potential confounders, for example: gender, age, and gaming experience. The primary outcomes were the experts’ score, the research team movement score and movement measured by an accelerometer. We hypothesized that more experienced sonographers make more goal-directed and efficient movements and that they would finish the exam faster. The research team counted the number of movements made. The experts scored predefined technical movements and mandatory visualizations for optimal IVC visualization (see appendix 1). We used a 4-point scale in which score 1 is bad, score 2 is moderate, score 3 is sufficient and score 4 is good. In other words: the higher the score the better the performance. The experts were two internists specialized in acute medicine as well as POCUS course instructors with both extensive ultrasound experience in real practice. They both reviewed the blinded movies separately, followed by a consensus score in case they scored items differently. The research team (consisting of 4 medical students) scored the exams in pairs (but independent from each other) according to a pre-developed scoring system as well (see appendix 2). They were also blinded for the two study groups and separately scored the exams. The average score of 2 researchers per participant was used for analysis. The checklist included the efficacy and quantity of hand/probe movements, the number of hints, the number of times the aorta and/or IVC had already been visualized before having been recognized by the participant, and total time. Finally, an accelerometer was attached to the 3D-printed probe to measure the exact movements in 3 different directions (horizontal, vertical, depth). After the experiment, a survey was completed to evaluate the game and to reflect on their skills in the ultrasound protocol.

### Statistical analysis

Statistical analysis was performed using IBM SPSS version 23. The variables in the categories were: hand movements, time and number of times the structures were visualized before being recognized as such. These variables were assessed using an independent t-test, as these were found to be normally distributed. The data from the experts’ opinion was assessed using a Mann-Whitney U test. Probe movement data from the accelerometer were added up from the three different directions and also divided by time (in seconds), composing the variable ‘total movement’. To assess the relationship between total movements from the accelerometer data and experts’ opinions, a linear regression was performed. For total amount of movements, the logarithm was used in regression analyses because of positive skew. Game data, including differences between the first and the second time level 1 was played, were assessed using a Mann-Whitney U test as data were not normally distributed. The significance of possible confounders was assessed using a Mann-Whitney U test. Fisher’s exact test was used for identifying gender as a possible confounder between groups. Data were reported as a mean and standard deviation (SD), or as a mean rank, where applicable. A *p*-value of < 0.05 was considered significant and a confidence interval (CI) of 95% was used if applicable. Benjamini Hochberg was used to correct for multiple comparisons and reduce the risk of type I error.

## Results

**Fig. 2:**
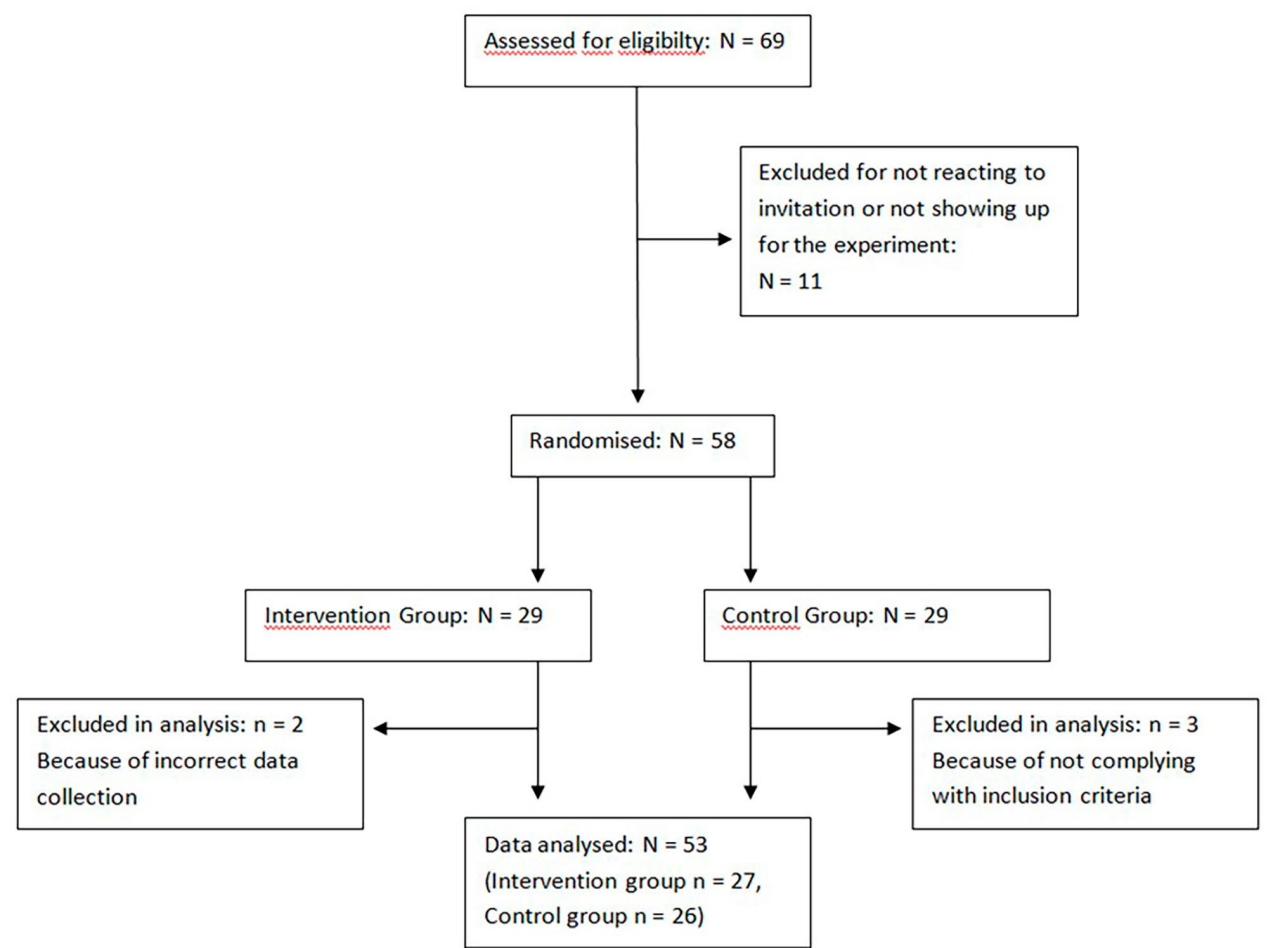
Study flow chart

After the invitation, 69 of 256 medical students responded to the Google Forms, of which 64 were scheduled due to the limited time span. Of these, 6 students did not show up resulting in a study population of 58 participants. Another 5 students were excluded from analysis due to invalid research data or previous ultrasound experience as stated in the questionnaire. A total of 53 students were included in the analysis, of which 27 students were placed in the intervention group and 26 in the control group (see Fig. 2).

Accelerometer data were obtained from 48 students as technical issues compromised the use of these data from five participants.

The mean age of participants was 21.4 years, with a predominance of female participants. There was no significant difference in gaming experience between the two groups (Table 1).

**Table 1:** Baseline characteristics

Participant characteristics	Total (N = 53)	Control (N = 26)	Intervention (N = 27)
female	42	18	24
male	10	7	3
other	1	1	
Mean age (years)	21.4	21.2	21.6
**Confounders**	***P*** **-value**	**Mean rank**	**Mean rank**
Assessment of gaming skills	0.482	28.40	25.65
Gaming experience 4–12 years	0.607	25.09	28.06
Gaming experience 12–18 years	0.306	29.17	24.91
Gaming experience > 18 years	0.639	27.98	26.06
Gaming experience last year	0.721	27.75	26.28
Gender	0.175		

The intervention group completed the exam faster (247 s vs. 347 s, *p* = 0.006 (95% CI: [30.20;168.80]) and lifted the probe less frequently from the model (0.54 vs. 3.79, *p* = 0.001 (95% CI: [1.39;5.11]) (Table 2). Participants in the intervention group also needed less hints to visualize the right structures (although not significant *p* = 0.077), while other probe movements did also not differ between the groups.

**Table 2:** Probe movement scores by researchteam

Scoring item	Control (N = 26) Number of times (SD)	Intervention (N = 27) Number of times (SD)	*P*-value
Lifting probe	3.67 (4.38)	0.54 (0.88)	**0.001**
Tilting to aorta *(number of tilting movements used until visualization of the aorta)*	12.15 (15.69)	10.96 (8.16)	0.729
Tilting to diameter aorta *(number of tilting movements used until visualization of the largest diameter of the aorta)*	2.77 (2.89)	4.07 (4.38)	0.221
Tilting to IVC *(number of tilting movements used until visualization of the IVC)*	5.10 (6.09)	3.56 (4.36)	0.295
Tilting to diameter IVC *(number of tilting movements used until visualization of the largest diameter of the IVC)*	1.98 (2.00)	1.41 (1.26)	0.218
Rocking to RA *(number of rocking movements used until visualization of the inlet of the RA)*	3.90 (3.41)	2.83 (2.54)	0.205
Total inefficient movements *(number of inefficient movements used until end of the protocol)*	19.69 (17.40)	17.09 (15.61)	0.577
Total time *(in seconds)*	347.40 (121.53)	247.91 (129.45)	**0.006**
Number of hints	5.60 (2.65)	4.22 (2.88)	0.077

Effect size (Cohen’s d) Lifting probe d ≈ 0.925 (large effect size), Total time d ≈ 0.773 (large effect size). (Table 3).

**Table 3:** Expert consensus score on different probe movements

Probe movements	Control (Mean rank)	Intervention (mean rank)	*P*-value
Probe holding	27.31	26.70	0.857
Aorta tilting	26.40	27.57	0.771
Aorta visualization	26.58	27.41	0.812
Aorta goal-oriented	26.23	27.74	0.706
IVC Tilting	24.04	29.85	0.142
IVC visualization	25.46	28.48	0.429
IVC goal-oriented	26.85	27.15	0.939
RA rocking	26.17	27.80	0.690
RA visualization	26.37	27.61	0.753
RA goal-oriented	25.69	28.26	0.529
General competence	25.10	28.83	0.361
Total score	25.71	28.24	0.550

The expert scores on probe movements and general performance did not differ between the two groups.

However, the intervention group made less movements, as measured by the accelerometer, than the other group (Table 4).

**Table 4:** Movement of probe as measured with accelerometer

Data type	Control (N = 23) (SD)	Intervention (N = 25) (SD)	*P*-value
Total amount of movement	1083.13 (543.80)	765.81 (339.21)	**0.018**
Movement/second	2.83 (0.92)	2.66 (0.66)	0.457

Effect size (Cohen’s d) total amount of movement d ≈ 0.646 (medium to large effect size)

**Fig. 3:**
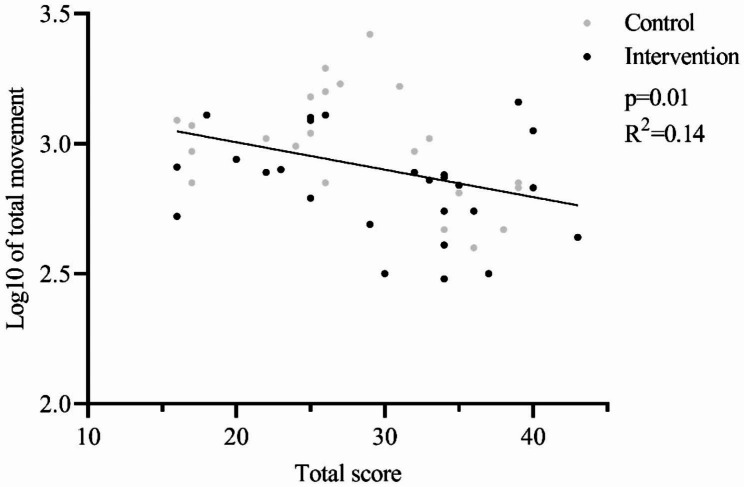
Linear regression analysis representing the relation between log total movements and total expert score

To further investigate these findings, additional analysis was carried out for the whole group, in which an inverse association was found between movements and the expert score (see Fig. 3).

### Learning curve of the game

We have recorded the change in gameplay parameters between the first and last time the participants played level 1 without having the participants being aware of this measurement. We found that all scores improved showing a 48% decrease in total playing time (*p* < 0.001), 36% reduction in attempts per coin (*p* = 0.007), a 33% reduction in total probe distance (*p* = 0.002), and a 61% decrease in contact moments (temporarily lifting the probe from the tablet, *p* < 0.001) (see supplementary file).

### Questionnaire

We inquired how the participants felt about the usefulness of the setup (Table 5).

**Table 5:** Questionnaire about usefulness of the setup ‘Underwater’ in learning ultrasound skills

Questionnaire item intervention group (27 participants)	Number (%)
Is the setup useful for training eye-hand coordination?	Bad 0 (0) Moderate 2 (7.4) Sufficient 12 (44.4) Good 13 (48.1)
Is the setup useful for training probe stability?	Bad 0 (0) Moderate 7 (25.9) Sufficient 8 (29.6) Good 12 (44.4)
Should serious games be used in the medicine curriculum?	Yes 12 (44.4) No 12 (44.4) No opinion 3 (11.1)
Is the setup useful to *simulate* probe handling?	Yes 26 (96.3.) No 0 (0) No opinion 1 (3.7)
Is the setup useful to *learn* probe handling?	Yes 25 (92.6) No 2 (7.4) No opinion 0 (0)
How much fun is the game to play?	No fun 0 (0) little fun 1 (3.7) fun 8 (29.6) very fun 18 (66.7)
Did playing the game improved your ultrasound skills?	Little 1 (3.7) Moderate 3 (11.1) Much 16 (59.3) Very much 7 (25.9)
When should the serious game be used in the training program?	
- Before practical training - Beside practical training, for novices - Beside practical training for advanced learners	Yes 17 (63.0) / No 10 (37.0) Yes 19 (70.4) / No 8 (29.6) Yes 0 (0%) / No 27 (100.0)

The majority of the participants indicated that the setup is useful for training eye-hand coordination and probe holding stability, as well as for learning and simulating probe handling. Moreover, most participants enjoyed playing the game and felt that it improved their ultrasound skills. They indicated that the optimal moment to play the serious game is before or during the practical sessions. None of the participants was of the opinion that the current setup is useful for advanced ultrasound learners.

## Discussion

Our study shows that the serious game ‘Underwater’ enhances ultrasound learning. The intervention group completed the exam faster and with less probe lifting during the ultrasound exam. Although the expert score did not differ between the two groups, the accelerometer showed less probe movements in the intervention group. Analysis of the whole group showed an inverse relationship between expert scores and probe movements. The game parameters (for example total playing time, attempts per coin, and contact moments) show increasing skills of the participants as demonstrated by the difference between the first and last level they have played. This indicates the learning effect of the game in probe handling. Finally, the participants positively value the use of this serious game to learn ultrasound and they feel it will help them in acquiring ultrasound skills. They also state that it is a fun alternative way to learn new skills and should therefore be used in ultrasound curricula. These results show that serious games, our research the game “Underwater” are useful for the enhancement of mastering medical technical skills.

We did not find any differences between the two groups in ultrasound performance regarding the expert scores. This may be explained by the fact that subtle improvements are difficult to identify when looking at all parameters at the same time, or due to the limitations of the video recording with a mobile device. Another explanation might be that subtle movements did not greatly improve the final ultrasound image, and hence there was no change in expert scores. It is also possible that the gaming time was too short to improve the spatial orientation and hand-eye-coordination skills. In our experience, it generally takes several hours or even longer to learn and improve probe movement skills, suggesting 30 min of playing time might not have been enough. Finally, the short ultrasound introduction for both groups might have been insufficient for performing a real ultrasound exam, limiting the possibility for the game to make a large difference. In future research, different applications and scanning protocols may be used with longer gaming time in advance.

To master ultrasound probe movements, several stages of learning may be identified as suggested by Fitts and Posner in 1967 [16]. The first stage is the cognitive stage where learners intellectualize and understand the task they need to master. In this stage, it might be important to limit the complexity of the movements and the amount of instructions. They should learn know about the effects of different ultrasound probe movements one at the time before integration of movements can occur. Also, they should develop a 3D mental image of the 2D screen and be able to predict which movement is needed to obtain or enhance a certain image. This relates to Jeannerod’s motor simulation theory, in which performing a motor action and thinking of doing that action activate similar motor systems in the brain [17]. Sufficient time should be allocated to practice with timely feedback of the performance. After this repetitive training and feedback, the learner reaches the next phase, the associative stage. In this stage the acquired knowledge is transferred to appropriate probe movements. The motor task will be more smoothly executed with fewer interruptions but active thinking to guide and predict the appropriate movements is still needed. Deliberate practice with feedback is the cornerstone of learning motor skills as suggested by Ericsson’s deliberate practice model [18]. In the final autonomous stage, the task is performed like the level of an expert, in an automated manner with minimal mental effort. In conclusion, acquiring ultrasound skills consisting of complex probe movements and creating a 3D mental model may take serious practice time, depending on the student. Ideally, this would take place under direct supervision of an ultrasound expert, but this takes precious time, effort, and money. Several studies show that lack of supervised practice time is one of the most important barriers for learning ultrasound [6, 7, 19–21]. This barrier may partially be overcome by using serious games as an alternative for developing ultrasound skills, which may reduce supervised bedside practice time although we have not investigated this effect in our study. We show that playing a serious game is a fun and useful additive to enhance learning performing ultrasound exams, improving precision, speed and reducing unnecessary probe movements. The advantage is that students can play as long as they want, at a time and place of their own choosing, and without supervision before practicing on real patients, which may overcome barriers in learning ultrasound.

Serious games are being developed and used to teach medical technical skills, but most games have not demonstrated improved skills in real practice, in contrast to the game ‘Underwater’ used in our study [9]. There is one publication about serious games for learning ultrasound skills and this concerns ultrasound-guided needle placement skills [22]. However, the setup is a simulator with some game elements rather than a serious game, and improvement of skills was only measured with the same setup, and not in real practice. Our game setup is relatively simple with a 3D printed probe and a tablet, and the software can be installed on personal or institutional laptops. The different game levels and layout can be changed extensively and possibly with adaptive learning strategies, enhancing ultrasound teaching. Finally, game development can incorporate several helpful functions to assist the student in learning, by showing the correct angle and movement. In this way the students receives instant feedback of his/hers performance but also suggestions how to improve the probe movements to fulfill the task better. Also, analysis of the multiple playing rounds may show structural errors leading to personalized learning objectives. By changing the game design with increasing complexity and difficulty, the game may also be suited for the more experienced sonographer.

There are several ultrasound simulators available which may also serve as an alternative way of learning ultrasound. One important disadvantage is the high costs of such a device, and most simulators use real medical simulation and not a game lacking the fun part of gaming.

### Limitations

We were not able to determine a sample size prior to this study as the effect size was not known. It is possible that several parameters or scores were not significant due to the relatively small sample size (N = 53), for example the expert scores for performance or the number of hints that were needed (*p* = 0.077). Recruitment of students was done by various WhatsApp groups. This may introduce a selection bias for more ultrasound or serious games enthusiast students. The intervention group played the game for 30 min, in contrast to the control group who directly performed the ultrasound after the introduction course. This may have induced a time-on-task bias, although the task “serious gaming” is different from performing a real ultrasound and the study goal was to investigate the effect of playing a serious game on real task performance. There is no standardized checklist for assessing ultrasound skills in research. A validation of our scoring system was not part of our study. We have developed the checklist with ultrasound experts based on their clinical ultrasound experience. The scoring of video’s is difficult and could induce interrater differences. We were able to calculate the interrater reliability (ICC) for the research team which were all above 0.8 (indicating good reliability) except for tilting of IVC (ICC 0.60). The ICC for the expert scores could not be determined but their scores were not significantly different between the intervention and the control group.

## Conclusion

The serious game ‘Underwater’ is a fun and useful addition to traditional bedside ultrasound learning, which also may overcome one of the most important barriers in learning ultrasound: lack of supervised practice time. We show that the game improves real-practice probe handling with faster and more goal-oriented probe movements. It uses a simple setup that can be used by students at their own pace and at any location, even at home.

### Electronic supplementary material

Below is the link to the electronic supplementary material.

Appendix 1: Scoring system for experts

Appendix 2: Scoring system of probe movements

## Data Availability

The datasets generated and/or analysed during the current study are not publicly available due to multiple datasets in foreign language (Dutch), but are available from the corresponding author on reasonable request.
