# Correction: Teaching point-of-care ultrasound using a serious game: a randomized controlled trial

**DOI:** 10.1186/s12909-024-05064-3

**Published:** 2024-01-25

**Authors:** Tycho Olgers, Jelle Rozendaal, Sanne van Weringh, Rachel van de Vliert, Ranek Laros, Hjalmar Bouma, Jan C. ter Maaten

**Affiliations:** 1 Department Internal Medicine, University of Groningen, University Medical Center Groningen, 9700 RB Groningen, The Netherlands; 2 Faculty of medical sciences, University of Groningen, Postbus 72, 9700 AB Groningen, The Netherlands

**Correction: BMC Medical Education (2023) 23:977**

10.1186/s12909-023-04964-0

Following publication of the original article [1], the authors identified an error in the author name of Jan C. ter Maaten.

The incorrect author name is: Jan ter Maaten.

The correct author name is: Jan C. ter Maaten.

## References

[1] Olgers, et al. BMC Med Educ. 2023;23977. 10.1186/s12909-023-04964-0.

