# Supplementary material for: Teaching point-of-care ultrasound using a serious game: a randomized controlled trial
Source: BMC Med Educ. 2023 Dec 19;23:977. doi: 10.1186/s12909-023-04964-0 (PMC10731722; doi:10.1186/s12909-023-04964-0)
Supplement: Supplementary file 1 — Appendix 1: Scoring system for experts [file 12909_2023_4964_MOESM1_ESM.docx]

Appendix 1. Scoring system for experts

| **Name of video, X:** | **Scored by:** | **Date:** |
| --- | --- | --- |

**Experts’ opinion (highlight or circle the best option)**

1. How well does the candidate hold the probe (logical position of hands, relaxed grip)?

++ + - --

(very good) (very bad)

1. How well does the candidate move the probe from the left abdomen to the aorta (far enough to the left, tilting, not too rigorous and aggressive)?

++ + - --

(very good) (very bad)

1. How well does the candidate visualize the aorta (maximum diameter and length)?

++ + - --

(very good) (very bad)

1. How goal-oriented (calm and logical vs. a lot and random, the right directions) were the candidate's movements for visualising the aorta?

++ + - --

(goal-orientated) (not goal-orientated)

1. How well does the candidate move the probe from the aorta to the IVC (far enough to the left, tilting, not too rigorous and aggressive)?

++ + - --

(very good) (very bad)

1. How well does the candidate visualize the vena cava (maximum diameter, wall visible, remains visualised during collapse/inspiration)?

++ + - --

(very good) (very bad)

1. How goal-oriented (calm and logical vs. a lot and random, the right directions) were the candidate's movements for visualising the IVC?

++ + - --

(goal-orientated) (not goal-orientated)

1. How good is the rocking motion to see the inlet of the IVC into the right atrium (enough but not too much rocking, enough pressure)?

++ + - --

(very good) (very bad)

1. How well does the candidate visualize the inlet of the vena cava into the right atrium? (enough of atrium visualised, heart valve visible, IVC still visible)

++ + - --

(very good) (very bad)

3

1. How goal-oriented (calm and logical vs. a lot and random, the right directions) were the candidate's movements for visualising the inlet of the IVC into the right atrium?

++ + - --

(goal-orientated) (not goal-orientated)

1. To what extent do you consider the candidate competent when making an ultrasound? (in general)

++ + - --

(able) (not able)

1. In your opinion, what should the candidate do differently next time?

______________________________________________________________

______________________________________________________________

______________________________________________________________

______________________________________________________________

______________________________________________________________

______________________________________________________________
