# Supplementary material for: Teaching point-of-care ultrasound using a serious game: a randomized controlled trial
Source: BMC Med Educ. 2023 Dec 19;23:977. doi: 10.1186/s12909-023-04964-0 (PMC10731722; doi:10.1186/s12909-023-04964-0)
Supplement: Supplementary file 2 — Appendix 2: Scoring system of probe movements [file 12909_2023_4964_MOESM2_ESM.docx]

Appendix 2. Scoring system of probe movements

| **What** |  | **Score** |
| --- | --- | --- |
| **Time** |  |  |
| Time before visualizing Aorta (from left abdomen positioning) (count from moment task is given) |  | In seconds |
| Time before visualizing largest diameter of Aorta (count from moment task is given) |  | In seconds |
| Time before visualizing Vena cava (count from moment task is given) |  | In seconds |
| Time before visualizing largest diameter of Vena cava (count from moment task is given) |  | In seconds |
| Time before visualizing Vena cava entering the right atrium (count from moment task is given) |  | In seconds |
| How many times has the Aorta already been on the screen before identified as certain by participant? |  | Number |
| How many times has the Vena cava already been on the screen (after aorta has been identified) before identified as certain by participant? |  | Number |
| **Hand movements** |  |  |
| Amount of tilting movements towards aorta in order to visualize aorta (from left abdomen positioning) (Count midline) |  | Tilting: |
| Amount of tilting movements towards the largest diameter of the aorta (Count midline) |  | Tilting: |
| Amount of tilting movements from visualizing the aorta to visualizing IVC (Count midline) |  | Tilting: |
| Amount of tilting movements towards the largest diameter of the IVC |  | Tilting: |
| Amount of rocking movements from visualizing the IVC to the IVC entering the right atrium |  | Rocking: |
| Amount of inefficient hand movements which didn’t contribute to visualizing structure   - From beginning - aorta optimal visualisation (amount of movements) - From aorta optimal visualisation - IVC optimal visualisation   (amount of movements)   - From IVC - right atrium   (amount of movements)  Inefficient: turning, sliding |  | Number  ….  ….  …. |
| Amount of lifting up the probe and starting over (when ultrasound screen is unclear) |  | Number |
| Amount of tips given (count each tip as a point also when mentioned multiple times, except for “apply more pressure” which is counted only once) |  | Number |
